# Cross-Linking Effects Dictate the Preference of Galectins to Bind LacNAc-Decorated HPMA Copolymers

**DOI:** 10.3390/ijms22116000

**Published:** 2021-06-01

**Authors:** Sara Bertuzzi, Ana Gimeno, Ane Martinez-Castillo, Marta G. Lete, Sandra Delgado, Cristina Airoldi, Marina Rodrigues Tavares, Markéta Bláhová, Petr Chytil, Vladimír Křen, Nicola G. A. Abrescia, Ana Ardá, Pavla Bojarová, Jesús Jiménez-Barbero

**Affiliations:** 1 CIC bioGUNE, Basque Research and Technology Alliance, BRTA, Bizkaia Technology Park, 48162 Derio, Bizkaia, Spain; sbertuzzi@cicbiogune.es (S.B.); a.m.gimenocardells@uu.nl (A.G.); amcastillo@cicbiogune.es (A.M.-C.); mgutierrez@cicbiogune.es (M.G.L.); sdelgado@cicbiogune.es (S.D.); nabrescia@cicbiogune.es (N.G.A.A.); aarda@cicbiogune.es (A.A.); 2 BioOrgNMR Lab, Department of Biotechnology and Biosciences, University of Milano-Bicocca, Piazza della Scienza 2, 20126 Milano, Italy; cristina.airoldi@unimib.it; 3 Institute of Macromolecular Chemistry of the Czech Academy of Sciences, Heyrovského Nám. 2, 16206 Prague, Czech Republic; tavares@imc.cas.cz (M.R.T.); mblahova@imc.cas.cz (M.B.); chytil@imc.cas.cz (P.C.); 4 Institute of Microbiology of the Czech Academy of Sciences, Vídeňská 1083, 14220 Prague, Czech Republic; kren@biomed.cas.cz; 5 Ikerbasque, Basque Foundation for Science, 48013 Bilbao, Bizkaia, Spain; 6 Department of Health Care Disciplines and Population Protection, Faculty of Biomedical Engineering, Czech Technical University in Prague, Nám. Sítná, 27201 Kladno, Czech Republic; 7 Department of Organic Chemistry II, University of the Basque Country UPV/EHU, 48940 Leioa, Bizkaia, Spain

**Keywords:** galectin, multivalency, glycomimetic, molecular recognition, HPMA copolymer, inhibition, glycopolymer

## Abstract

The interaction of multi-LacNAc (Galβ1-4GlcNAc)-containing *N*-(2-hydroxypropyl) methacrylamide (HPMA) copolymers with human galectin-1 (Gal-1) and the carbohydrate recognition domain (CRD) of human galectin-3 (Gal-3) was analyzed using NMR methods in addition to cryo-electron-microscopy and dynamic light scattering (DLS) experiments. The interaction with individual LacNAc-containing components of the polymer was studied for comparison purposes. For Gal-3 CRD, the NMR data suggest a canonical interaction of the individual small-molecule bi- and trivalent ligands with the lectin binding site and better affinity for the trivalent arrangement due to statistical effects. For the glycopolymers, the interaction was stronger, although no evidence for forming a large supramolecule was obtained. In contrast, for Gal-1, the results indicate the formation of large cross-linked supramolecules in the presence of multivalent LacNAc entities for both the individual building blocks and the polymers. Interestingly, the bivalent and trivalent presentation of LacNAc in the polymer did not produce such an increase, indicating that the multivalency provided by the polymer is sufficient for triggering an efficient binding between the glycopolymer and Gal-1. This hypothesis was further demonstrated by electron microscopy and DLS methods.

## 1. Introduction

Galectins are carbohydrate-binding lectins (15 members in humans) characterized by a carbohydrate recognition domain (CRD) responsible for affinity to terminal β-galactoside glycostructures [1]. This lectin family possesses a number of biological activities related to the development and progression of cancer, which is primarily associated with galectin-1 (Gal-1) and galectin-3 (Gal-3). Both Gal-1 and Gal-3 are prominent cancer-related galectins, and their expression levels have been found to be dysregulated in various cancer cells and tissues [2,3,4]. In particular, they participate in cellular adhesion, invasion, angiogenesis, and metastatic processes, thus supporting the development and spread of tumors [5]. Their ability to induce apoptosis of T cells leads to the simultaneous suppression of the immune system during cancerogenesis [6]. For these reasons, Gal-1 and Gal-3 are recognized as prospective targets for therapeutical inhibition, and the search for their potent inhibitors is a widely developed line of biomedical research [7,8].

The structure and architecture of the CRD of galectins strictly determine their carbohydrate-binding specificity. Whereas Gal-1 is an electrostatic dimer of two identical CRD units, Gal-3 oligomerizes through its non-CRD N-terminal portion. Besides natural ligands such as lactose, LacNAc (Galβ4GlcNAc), and LacdiNAc (GalNAcβ4GlcNAc), both galectins have shown affinity to epitopes based on (multi-)*N*-acetyllactosamine, lactulose, blood-group oligosaccharides, and other structures, including glycomimetics [9,10,11]. Carbohydrate-protein interactions are intrinsically weak, usually in the mM-µM for monovalent interactions. Nevertheless, lectins are fairly specific and usually recognize only one or two given monosaccharide entities. To achieve the required biological responses, lectins bind multivalent presentations of glycan chains located on the surface of the cells. Therefore, the use of multivalent carbohydrates to address molecular recognition problems in glycosciences provides hints on the phenomena that take place in the cell environment. Indeed, the multivalent presentation of carbohydrate ligands in a range of carriers, such as proteins, oligonucleotides, fullerenes, calixarenes, dendrimers, and nanoparticles, has been described. This approach has demonstrated that the affinity of the corresponding interaction can increase by several orders of magnitude with respect to the monovalent ligand [12,13,14,15,16].

**Figure 1:**
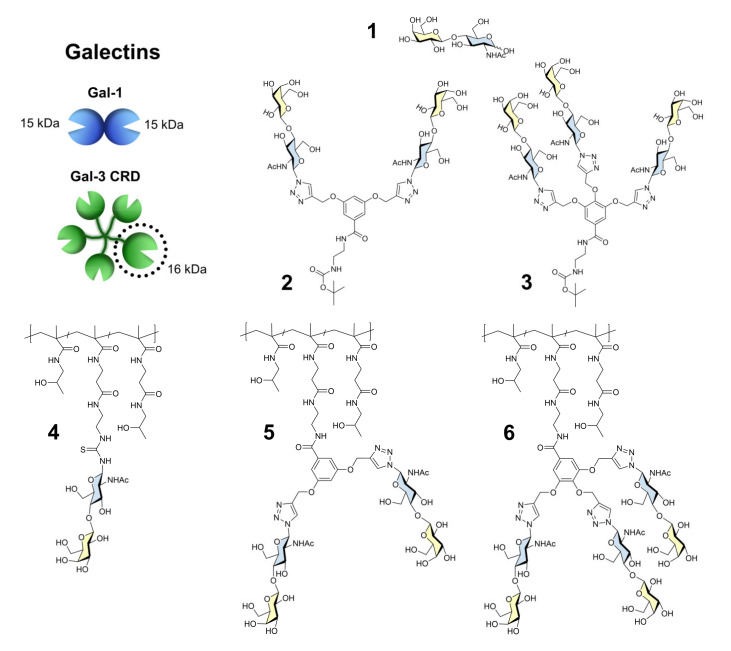
Structural representation and numeration of the ligands **1**–**6** whose interaction with Gal-1 and Gal-3 CRD was studied in this work.

In this work, we employed ligand-based (STD-NMR) and receptor-based (^1^H-^15^N HSQC) NMR methods to examine, with atomic resolution, the intermolecular interaction of Gal-1 and Gal-3 CRD with a series of five multivalent compounds that display different structural scaffolds. The ligands tested as putative inhibitors of galectin-mediated interactions presented a common LacNAc (Galβ4GlcNAc) motif (Figure 1) [17,18,19].

STD-NMR experiments provide information on the existence of interaction and on the precise ligand binding epitope, while ^1^H-^15^N HSQC spectra make it possible to assess the interaction, as well as deduce the protein binding site, the existence of exchange phenomena, and the possible generation of larger-order complexes. Therefore, to provide a full perspective of the interaction, the NMR-based analysis of the binding events was complemented with additional experimental evidence at a different size scale through cryo-electron microscopy (cryo-EM) experiments and dynamic light scattering (DLS). 

Indeed, the NMR spectra provided clear hints on the generation of large supramolecular structures between the galectin-1 and the glycopolymers. Moreover, Gal-1 generated large supramolecular entities through cross-linking. This fact has been unequivocally demonstrated by the DLS and especially the EM experiments, illustrating that the combination of complementary methods and protocols (NMR, DLS, EM) affords a clear representation of the molecular recognition process. 

The employed multivalent systems were specifically designed and contain synthetic water-soluble carriers based on *N*-(2-hydroxypropyl) methacrylamide (HPMA) copolymer. This polymer is attractive for in vivo applications for its biocompatibility, good water solubility, prolonged circulation time, specific delivery to biological targets, and lack of toxicity or immunogenicity. In addition, the synthesis of well-defined HPMA carriers containing accurate amounts of several functionalities has been described, enabling further attachment of bioactive compounds [20,21]. Nevertheless, the design of HPMA in terms of polymer architecture, molecular weight, and spacer structure, as well as the mode of presentation of the glycans, may strongly influence the lectin binding event and, eventually, the associated biological activity [22,23,24].

## 2. Results and Discussion

### 2.1. Synthesis of Ligands ***2***–***6***

The standard comparative ligand **1** (LacNAc; Galβ4GlcNAc) was obtained commercially. The functionalized LacNAc derivatives used for conjugation to multivalent carriers were synthesized by chemo-enzymatic procedure from respective functionalized GlcNAc acceptors by the action of β-galactosidase from *Bacillus circulans* from the commercial preparation Biolacta^®^ [25,26]. The synthesis of ligands **2**–**6** was performed as described in our earlier work [24]. The controlled radical RAFT (reversible addition−fragmentation chain transfer) polymerization technique was applied to prepare the polymer precursor poly(HPMA-*co*-MA-AP-TT) with a molecular weight of 24,600 g mol^−1^ and narrow dispersity (*Đ* ≈ 1.1). The copolymer contained 23 mol.% of reactive thiazoline-2-thiol (TT) groups statistically distributed along the polymer chain, which enabled the covalent attachment of the amino-functionalized LacNAc (19.3 mol.%) and afforded the glycopolymer with individual presentation (ligand **4**). For the clustered presentations, branched ligands containing either two or three propargyl moieties were first reacted with the azido-functionalized LacNAc via Cu(I)-catalyzed azide−alkyne cycloaddition (CuAAC), affording the glycosylated branched ligands in bivalent (ligand **2**) or trivalent (ligand **3**) presentation which still contained a *t*-Boc group protecting the primary amino group. The carbohydrate conjugation was proved by the appearance of the triazole moiety signal in the ^1^H NMR spectrum. The subsequent *t*-Boc deprotection was performed under acidic conditions. Aminolysis of the revealed primary amines with the TT groups of the polymer precursor was performed, yielding the glycopolymers carrying bi- (ligand **5**) or trivalent (ligand **6**) branching, which contained 20.9 mol.% and 22 mol.% of LacNAc, respectively. The carbohydrate conjugation resulted in higher molecular weights of all glycopolymers compared to their polymer precursor. In addition, the dispersity slightly increased due to the statistical distribution of LacNAc along the polymer chain. Even maintaining a comparable content of LacNAc, the introduction of bi- and trivalent branching led to a higher molecular weight than the individual presentation. The physicochemical characteristics of glycosylated branched ligands **2** and **3** as well as glycopolymers (ligands **4**–**6**) are shown in Table 1.

### 2.2. Inhibitory Activity Evaluation

Ligands **1**–**6**, all containing the same carbohydrate structure of LacNAc, were assayed as inhibitors of binding of Gal-1 or Gal-3 to asialofetuin in a competitive ELISA-type assay (Table 2). For the aim of this study, we selected two small-molecule branched ligands **2** and **3** (bi- and trivalent, respectively), and three glycopolymer ligands with a comparable LacNAc content (19–22 mol.%) but with a different LacNAc presentation (**4**, individually distributed LacNAc; **5**, LacNAc on bivalent branching; and **6**, LacNAc on trivalent branching). As extensively discussed in our previous work, significant differences were found for binding to either galectin depending on the ligand structure. In the case of small-molecule ligands **2** and **3**, a higher inhibitory potency was found for the trivalent ligand **3** for both galectins. When comparing three glycopolymers with a comparable sugar content (19–22 mol.%), both galectins behaved quite differently. Gal-3 exhibited a relatively slight increase in inhibitory potency with the glycopolymer ligands **4**–**6** in either presentation. Though it slightly preferred LacNAc clustered presentation on bi- and especially trivalent branching (ligands **5** and **6**, respectively) on the polymer, the overall differences in affinity did not exceed one order of magnitude compared to the small-molecule ligands **2** and **3**. The situation was completely different for Gal-1. Gal-1 strongly preferred glycopolymer ligands **4**–**6**, exhibiting an increase in affinity of up to two orders of magnitude compared to small-molecule ligands **2** and **3**. The bivalent clustered presentation of glycopolymer **5** was the least preferred structural arrangement of LacNAc, ca five times worse than the individual presentation (**4**) and the trivalent presentation (**6**). All glycopolymer ligands **4**–**6** had inhibitory potency (*IC*_50_ values) in the nanomolar range (Table 2).

### 2.3. Molecular Recognition

In order to obtain detailed structural information regarding the binding of Gal-3 and Gal-1 to the ligands under study (**1**–**6**), a combination of NMR, DLS (Dynamic Light Scattering), and microscopy protocols was adopted [27]. The understanding of the complex interactions between the lectins and the polymeric ligands (**4**–**6**) was managed using a reductionist approach, starting from the study of single building blocks containing either two or three LacNAc moieties (**2**–**3**).

#### 2.3.1. NMR Studies

The molecular recognition process has been comprehensively investigated using NMR protocols, both from the viewpoint of the ligand (saturation-transfer difference NMR, STD-NMR) and from the viewpoint of the protein (^1^H-^15^N Heteronuclear Single Quantum Coherence NMR; HSQC) using the ^15^N-labelled galectins [27].

Due to the substantial intrinsic difference between the individual building blocks (**2**–**3**) and the polymeric ligands (**4**–**6**), ad hoc strategies were adopted for the analysis of the two types of systems.

**Figure 2:**
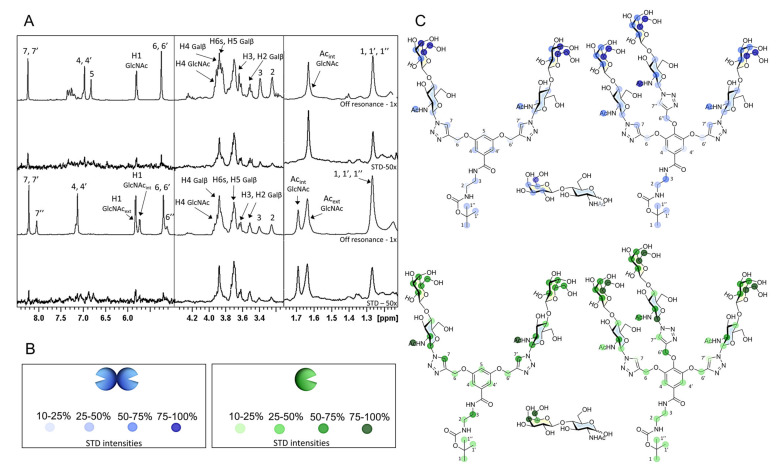
^1^H-STD-NMR results. (**A**) ^1^H STD-NMR spectra of the mixtures of Gal-1 (100 µM) with compounds **2** and **3** using a lectin/ligand molar ratio of 1:30 and 2 s of saturation time. From top to bottom: Off-resonance spectrum with annotations of the main ^1^H signals showing STD for the complex Gal-1/**2**; STD spectrum obtained upon irradiation at δ −0.5 ppm; off-resonance spectrum with annotations of the main ^1^H signals showing STD for the complex Gal-1/ligand **3**; STD spectrum obtained upon irradiation at δ −0.5 ppm. (**B**) Color legend of the representation of the relative STD intensities for each lectin. (**C**) Epitope mapping derived from the ^1^H-STD-NMR for the interaction of human Gal-1 (blue) and Gal-3 (green) with **1**, **2**, and **3**.

STD-NMR experiments were optimized and exploited to obtain the binding epitopes of ligands **1**–**3** toward human Gal-1 and Gal-3 (Figure 2).

All experiments were acquired at 298 K and with different ligand/lectin ratios depending on the type of ligand to optimize the STD signals. In fact, for each ligand/protein sample, the on-resonance saturation frequency at the aliphatic region was set at three different values: 0 ppm, −0.5 ppm, and −1 ppm.

The absolute STD (STD-AF) values were evaluated for the NMR signals of the ligand and the proton signal with the strongest STD effect was used as reference. Consequently, the relative STD intensities (STD%) were calculated, allowing us to map the ligand-binding epitope (Figure 2, panels B and C) [28,29,30].

Overall, the results show the existence of specific interactions of ligands **2** and **3** with both human Gal-1 and Gal-3. In particular, significant STD signals detected for **2** and **3** (medium H2 and H3, medium-strong H4, strong H5, and H6 of the Galβ unit), which perfectly match the binding epitope for the simple LacNAc unit (**1**) and, in general, the typical pattern of the interaction of galectins with β-Gal-containing moieties [8]. Additional strong signals were detected for the acetyl of GlcNAc moiety. Moreover, some protons of the scaffold displayed STD (Figure 2, panel A) due to predictable transient contacts of the whole structure with the surface of the protein. 

Comparing all the experiments, it is possible to affirm that the main binding epitope is the β-Gal ring of the disaccharide for both Gal-1 and Gal-3 and that the multiple sugar presentation does not modify the recognition. 

It is interesting to note that the experiments performed with Gal-1 are of a considerably better quality in terms of STD signal intensities than those performed with Gal-3. The explanation of this phenomenon probably lies in the different sizes of the two proteins, which makes the Gal-1 dimer (*M*_W_ = 30 kDa) a better vehicle for irradiation transmission than monomeric Gal-3 (*M*_W_ = 16 kDa) (Table 2).

Moreover, for **2** and **3**, the STD results obtained after irradiation at different aliphatic frequencies (δ of 0.00 ppm, −0.5 ppm, and −1 ppm) were compared (Supplementary Figures S1–S5). 

With respect to Gal-1, the absolute intensities displayed a slight systematic variation among irradiations caused by a change in the efficiency of the irradiation of the protein. Nevertheless, the relative intensities remain constant with the different irradiations. These observations reflect the robustness of the binding epitope obtained. For Gal-3, although the comparison displays larger variations, the ligand epitope remains very clear. 

The interaction was also monitored from the lectin perspective. Thus, a series of ^1^H-^15^N HSQC NMR experiments were performed where increasing amounts of ligands **1**–**3** were added to samples containing ^15^N labeled either Gal-1 or Gal-3 protein. Since the binding of the ligand modifies the chemical shifts of the amide signals of the protein, it is possible to rationalize the observed Chemical Shift Perturbations (CSP) in the protein signals to obtain structural information of the molecular recognition event [31]. 

**Figure 3:**
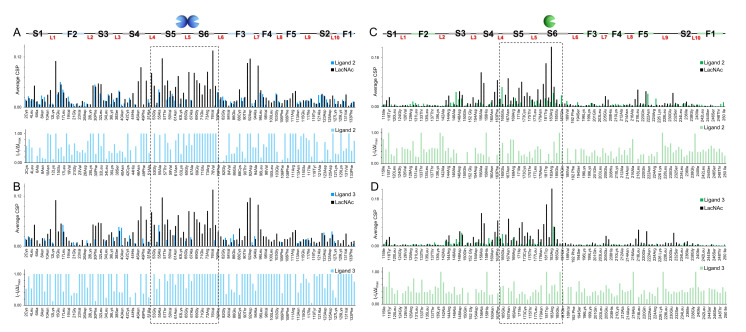
CSP and intensity analysis. (**A**) Above: CSP plot obtained for the complex Gal-1/**2** (in light blue) compared to that for Gal-1/**1** (in black); below: Intensity-loss plot obtained for Gal-1/**2.** (**B**) Above: CSP plot obtained for Gal-1/**3** (in light blue) compared to Gal-1/**1** (in black); below: Intensity-loss plot obtained for Gal-1/**3**. (**C**) Above: CSP plot obtained for Gal-3/**2** (in green) compared to Gal-3/**1** (in black); below: Intensity-loss plot obtained for Gal-3/**2**. (**D**) Above: CSP plot obtained for Gal-3/**3** (in green) compared to Gal-3/**1** (in black); below: Intensity-loss plot obtained for Gal-3/**3**.

Fittingly, the CSP plots obtained for Gal-1 and Gal-3 in the presence of **1** correspond to the profile for these interactors (Figure 3). Only the canonical binding site of the lectin was perturbed (mainly strands S5 and S6) [8,17,18].

Interestingly, a different behavior was observed for the interactions with ligands **2** and **3**: A significant reduction of the intensities of the ^1^H-^15^N HSQC cross-peaks of both lectins was detected during these titrations, especially when Gal-1 was employed. The statistical rebinding event that occurred due to the bivalent or trivalent presentation of the ligands, combined with the free-bound chemical exchange process, produced an important increase in the relaxation rate of the NMR signals and, consequently, the decrease of the cross-peaks in the HSQC spectra.

To make semiquantitative comparisons, the HSQC spectra for the lectins in the presence of a similar number of equivalents (between 5 and 7.5) of LacNAc epitopes per active site were analyzed. Besides the CSP plots, the variations in the cross-peak intensities for every amino acid residue were also measured (Figure 3).

These intensity plots provide the perspective of those signals that completely disappeared during the titrations. From the analysis of all the plots, the most perturbed residues in the presence of **1** (CSP plot) were those that disappeared in the presence of **2** and **3** (Intensity plots), strongly suggesting that those residues are the key ones for binding in both cases (Figure 3). These shreds of evidence confirm that the recognition process of ligands **2** and **3** by the lectins is comparable to that observed for the simple LacNAc unit.

**Figure 4:**
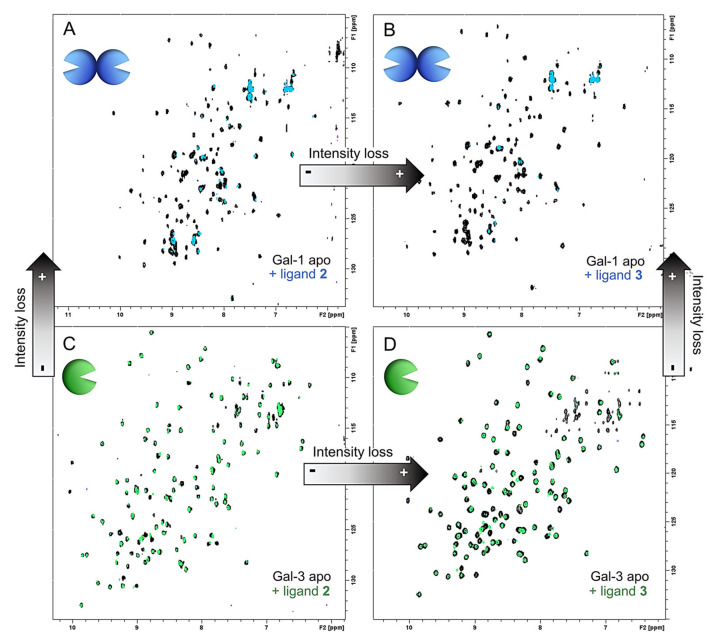
Comparison of the total relative (bound versus apo) loss of intensity of ^1^H-^15^N HSQC spectra. (**A**) Stacked ^1^H-^15^N HSQC spectra of Gal-1 apo (black) and Gal-1 upon addition of 5 equivalents (10 active equivalents) of **2** (light blue). (**B**) Stacked ^1^H-^15^N HSQC spectra of Gal-1 apo (black) and Gal-1 upon addition of 2.5 equivalents (7.5 active equivalents) of **3** (light blue). (**C**) Stacked ^1^H-^15^N HSQC spectra of Gal-3 apo (black) and Gal-1 upon addition of 5 equivalents (10 active equivalents) of **2** (green). (**D**) Stacked ^1^H-^15^N HSQC spectra of Gal-3 apo (black) and Gal-1 upon addition of 2.5 equivalents (7.5 active equivalents) of **3** (green).

Furthermore, the comparison of the total intensities measured in the HSQC spectra allowed us to extrapolate a general trend: The intensity loss is much more pronounced in the complexes with Gal-1 than with Gal-3, and it is always higher for the trivalent ligand **3** versus the bivalent **2** (Figure 4). The observed decrease of signal intensities can be explained by the transient formation of large supramolecular complexes that provide fast relaxation and, therefore, line broadening with the concomitant intensity loss. 

Notably, the NMR data support the hypothesis that, for Gal-3, the interaction with **2** and **3** occurs through a canonical mechanism at the typical binding site. The observed preference of Gal-3 for **3** (Table 2) can be justified by the triple presentation of epitopes that generate more favorable statistical rebinding effects than for **2**. 

On the contrary, the extended intensity reduction of the protein signals for Gal-1 indicates that a different type of interaction occurs. Very likely, the generation of supramolecular lectin-ligand complexes takes place as a combination of the multivalent presentation of **2** and **3** and the dimeric architecture of Gal-1 that favors the formation of larger aggregates through cross-linking effects.

Ligand-based STD NMR experiments were not attempted to deduce the details of the interactions of the galectins with the glycopolymers **4**–**6** given the large molecular weight of these molecules, which is beyond the limits of the experiment [28,29,30]. Alternatively, the CSP strategy with the labeled proteins was adopted to obtain information on the binding event from the protein point of view. However, the spectra acquired after the addition of small amounts of glycopolymers appeared empty, with an almost complete loss of the lectin cross-peaks, which was likely caused by the formation of supramolecular structures undetectable by NMR. The approach adopted to recover the protein signals after the addition of **4**, **5**, and **6** was a subsequent titration with a known competitor, the basic compound **1**. Obviously, if **4**, **5**, and **6** interact at the canonical lectin binding site, the addition of an excess of **1** should shift the equilibrium toward the formation of the lectin complexes with **1**. Therefore, the lectin cross-peaks should be visible again in the HSQC spectra.

**Figure 5:**
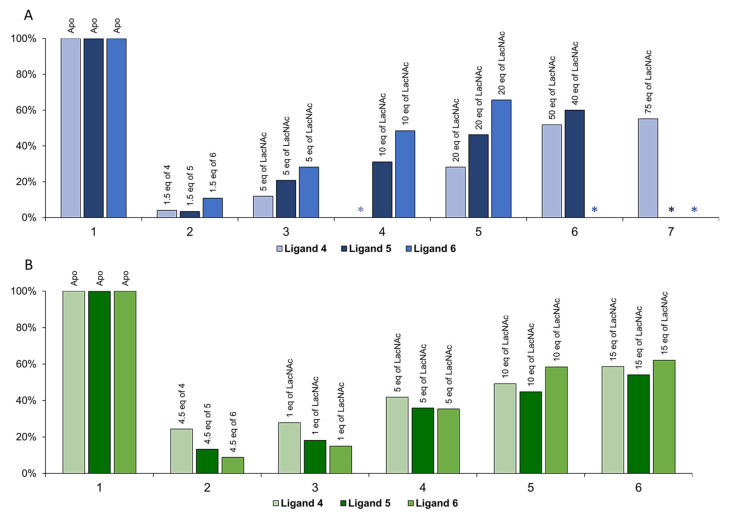
^1^H-^15^N HSQC competition experiments with ligands **4**–**6**, following the relative total intensity. Each ligand is represented with different shades of the same color (legend below each chart). Symbol * stands for spectra not acquired. (**A**) Histogram chart for the total relative intensity derived from HSQC spectra of Gal-1 apo (x-axis, group 1), Gal-1 with 1.5 equivalents of LacNAc epitopes per galectin site of ligands (**4**–**6**, group 2), and following signal recovery (groups 3–7 upon addition of 5, 10, 20, 40, and 75 equivalents of competitor **1**, respectively). (**B**) Histogram chart for the total relative intensity derived from HSQC spectra of Gal-3 apo (group 1), Gal-3 with 4.5 equivalents of LacNAc epitopes per galectin site of ligands (**4**–**6**, group 2), and following signal recovery (groups 3–6 with the addition of 1, 5, 10, and 15 equivalents of competitor **1**, respectively).

Fittingly, the cross-peaks were gradually recovered upon addition of increasing amounts of LacNAc. A high excess of the competitor enabled a partial dissociation of the lectin-glycopolymer complex, followed by the recovery of the HSQC signal intensities (Figure 5). The magnitude of the recovery should depend on the relative affinity of the lectin-ligand complexes.

The results obtained for Gal-1 (Figure 5, panel A) show that the total recovery of the signals was never achieved. Indeed, visual inspection of the NMR tube allowed us to detect the formation of insoluble species which could not be solubilized. In any case, markedly different behavior for ligands **4**–**6** was also evident. The addition of small amounts of glycopolymer **4** with individual LacNAc distribution (only 1.5 equivalents of LacNAc epitopes versus galectin binding site) caused a dramatic loss of signal (96%), strongly suggesting the presence of a cross-linking phenomenon. By adding five equivalents of competitor **1**, only 12% of the signal was recovered. The maximum recovery of the signal intensities (55%) was achieved using 75 equivalents of **1**. This indicates a fairly significant interaction between Gal-1 and ligand **4**.

A similar loss of signal was also detected after the addition of compound **5** to Gal-1. However, in this case, the signal recovery was faster compared to that observed with **4**: Upon adding 5 equivalents of **1**, 21% of signal intensities were recovered, and with 40 equivalents, the recovery reached 60%. Thus, the binding event was somewhat less efficient than in the case of the glycopolymer **4** with the same number of individually distributed LacNAc epitopes versus galectin added. The analysis of the data suggests that there are no cooperative effects promoted by the bivalent presentation of **5**. Indeed, the tendency is the opposite.

For glycopolymer **6**, even if the molar ratio between the available LacNAc units and Gal-1 was identical to that for **4** and **5**, the initial loss of signal was even less dramatic (89%). By adding 5 equivalents of **1**, 28% of the signal was recovered, while 66% of the original intensity was reached with only 20 equivalents. The binding was less efficient than that with **4**, which featured individual distribution. Again, the trivalent presentation of **6** did not promote cooperative effects and was even less favorable than the bivalent presentation. 

The loss of NMR signal intensities due to the interaction with the glycopolymers also occurred with human Gal-3 (Figure 5, panel B). However, the effect was less dramatic (76% for **4**, 87% for **5,** and 91% for **6**) when compared to the results obtained with Gal-1, although there were more LacNAc epitopes available per binding site (4.5 equivalents in each experiment). The binding was much less effective: No cross-linking effects occurred in this case, given the monomer character of the lectin. The trend was homogeneous for all the compounds, and there was not any cooperative effect between the multiple epitopes of **5** and **6**.

Globally, by comparing the obtained results, **4**, **5**, and **6** show higher affinity for the prototype Gal-1 than for Gal-3. According to the NMR data, the relative potency per active LacNAc unit is higher for **4** with respect to Gal-1, while Gal-3 does not display a clear preference for any of the three glycopolymers. This is in basic agreement with the affinity data acquired from the ELISA-type assay for Gal-1 (Table 2). Whereas the affinities of all tested glycopolymers to Gal-1 reached nanomolar range with a considerable avidity contribution, in the case of Gal-3, they ranged in micromolar range without any major avidity enhancement accomplished through the multivalent presentation. We suppose that more subtle affinity differences not exceeding one order of magnitude, which were documented in the ELISA assay, are not observable by the present NMR methods.

#### 2.3.2. DLS and Cryo-EM Measurements

To further explain the experimental pieces of evidence obtained from the NMR analysis and provide additional complementary information, Cryo-EM and DLS methods were employed. 

First, the size of the complex formed by the glycopolymer **4** (individually distributed LacNAc) with both galectins was estimated using DLS (Supplementary Figure S6). Initially, the biomolecules were measured separately. The measured hydrodynamic radius was 1.56 nm ± 0.19 for Gal-3, 4.54 nm ± 0.6 for Gal-1, and 7.47 nm ± 1.8 for **4**. Then, both galectins were mixed with **4** in a 5:1 (galectin/ligand) ratio (corresponding to 1.5 epitopes per Gal-1 binding site and 3 epitopes per Gal-3 binding site) in separate experiments. The hydrodynamic radius of the mixture with Gal-3 barely increased (8.43 nm ± 1.23) with respect to the biomolecules alone. In contrast, when adding Gal-1, the particle size dramatically increased to 875.0 nm ± 128.1, in agreement with the NMR-based expectations of the presence of supramolecules generated through cross-linking effects.

**Figure 6:**
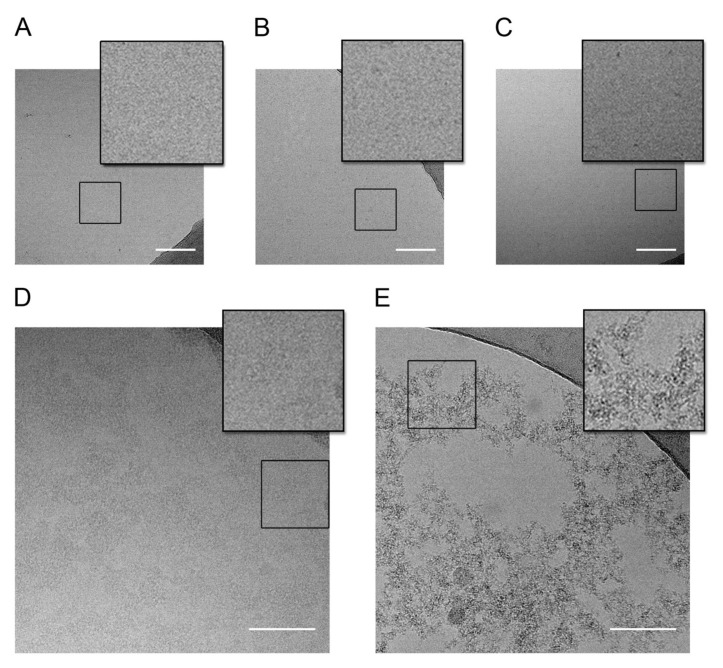
Cryo-EM images. (**A**) Gal-1 alone (0.5 mg/mL); (**B**) ligand 4 alone (0.17 mg/mL); (**C**) Gal-3 alone (0.5 mg/mL); (**D**) Gal-3 + ligand 4 (state concentration); (**E**) Gal-1 + ligand 4 (state concentration). Scale bar = 100 nm. The NMR lectin/ligand ratios were maintained.

Furthermore, the behavior of Gal-1 and Gal-3 in the presence of glycopolymer **4** was also investigated by cryo-EM. The analysis of the images acquired for the controls and the two mixtures indicated the existence of two distinguishable phenomena (Figure 6). First, all the controls for the isolated partners displayed a homogeneous distribution, and no aggregates were detected. In contrast, the formation of networks (or aggregation) was evident for the mixtures of **4** with Gal-3 and Gal-1. Fittingly, the images allowed us to assess that the tendency to form supramolecular complexes between Gal-1 and **4** is much more pronounced than for Gal-3.

## 3. Materials and Methods

Ligand **1** (LacNAc; Galβ4GlcNAc) was obtained from Carbosynth (Compton, UK). The preparation of β-galactosidase from *Bacillus circulans* Biolacta^®^ was from Daiwa Kasei (Aichi, Japan). *β*-Alanine, 2,2′-azobisisobutyronitrile (AIBN), 2-cyanopropan-2-yl dithiobenzoate, 2-thiazoline-2-thiol (CTA), CuBr, methyl 3,5-dihydroxybenzoate, methyl-3,4,5-trihydroxybenzoate, *N*-Boc-ethylenediamine, and *N*,*N*-diisopropylethylamine (DIPEA) were obtained from Sigma-Aldrich (Prague, Czech Republic). Propargyl bromide was purchased from Acros Organics (Pardubice, Czech Republic), and trifluoroacetic acid (TFA) was acquired from Iris Biotech (Marktredwitz, Germany).

### 3.1. Synthesis of Ligands ***2***–***6***

The synthesis of azido- and amino-functionalized LacNAc was performed as described previously [24]. The structural data obtained from ^1^H and ^13^C NMR experiments were in accordance with the structures. 

#### 3.1.1. Synthesis of Ligands **2** and **3**

The small-molecule glycosylated branched ligands in bivalent (ligand **2**) or trivalent (ligand **3**) presentation were synthesized from starting materials methyl-3,5-dihydroxybenzoate and methyl-3,4,5-trihydroxybenzoate, respectively, as previously described. First, propargylation was performed, followed by the methyl ester hydrolysis step. After amide formation using *N*-Boc-ethylenediamine, the branched ligands containing either two or three triple bonds were reacted with the azido-functionalized LacNAc via Cu(I)-catalyzed azide−alkyne cycloaddition (CuAAC) in DMF, affording the glycosylated branched ligands containing a *t*-Boc group protecting the primary amino group.

#### 3.1.2. Synthesis of Ligands **4**–**6**

The monomers HPMA and *N*-methacryloyl-*β*-alanine thiazolidine-2-thione (MA-AP-TT) were synthesized as previously reported [32,33]. Poly(HPMA-*co*-MA-AP-TT) polymer precursor was synthesized by controlled radical RAFT (reversible addition−fragmentation chain transfer) polymerization, using 2,2′-azobisisobutyronitrile (AIBN) and 2-cyanopropan-2-yl dithiobenzoate as initiators and CTA. Reaction conditions and the removal of the CTA group were shown before [24]. To yield the individual presentation of LacNAc (Ligand **4**), the amino-functionalized LacNAc was reacted with thiazoline-2-thiol (TT) functional groups of polymer precursors using DIPEA. In the case of glycopolymers carrying bi- (ligand **5**) or trivalent (ligand **6**) branching, the *t*-Boc protecting group was first removed from **2** and **3** under acidic conditions, then the primary amines were reacted with TT groups from the polymer precursor, as described recently [24].

### 3.2. Size Exclusion Chromatography

A Shimadzu HPLC system equipped with a size exclusion chromatography (SEC) column was used to determine the molecular weights (number-average molecular weight, *M*_n_, and weight-average molecular weight, *M*_w_), and dispersity (*Đ*) of ligands **4**–**6**. Measurements were performed using 0.3 M sodium acetate buffer (pH 6.5) as a mobile phase for Superose 6 (10 × 300 mm) at a flow rate of 0.5 mL min^−1^. Poly(HPMA-*co*-MA-AP-TT) polymer precursor used for preparing ligands **4**–**6** was measured in methanol/0.3 M sodium acetate buffer (pH 6.5, 4/1, *v*/*v*) using a TSKgel Super SW3000 (4.6 × 300 mm) at a flow rate of 0.3 mL min^−1^. A multiangle light scattering (MALS) detector (DAWN HELEOS II, Wyatt Technology Co., USA), Optilab-rEX differential refractometer index (RI) detector and SPD-M20A photodiode array detector (Shimadzu, Japan) were employed.

### 3.3. Ultraviolet−Visible Spectrophotometry

A Specord 205 ST spectrophotometer (Analytik Jena AG, Jena, Germany) was used to determine the content of TT groups in the polymer precursor, using *ε*(TT) = 10,300 L mol^−1^ cm^−1^ as molar absorption coefficient (λ_max_ = 305 nm) [32].

### 3.4. Competitive ELISA Assay

Recombinant His-tagged constructs of human Gal-1 and Gal-3 used in the ELISA-type assay were produced and purified as described previously [34]. The potential of ligands **1**–**6** to inhibit binding of human Gal-1 or Gal-3 to immobilized asialofetuin (ASF, Sigma Aldrich, Steinheim, Germany) was analyzed in a competitive ELISA assay. The F16 Maxisorp NUNC-Immuno Modules (Thermo Scientific, Roskilde, Denmark) were coated with ASF (0.1 μM, 50 μL/well) in PBS buffer (50 mM NaH_2_PO_4_/150 mM NaCl pH 7.5) overnight, followed by blocking with BSA (2% *w*/*v*) in PBS (1 h, r.t.). Then, increasing concentrations of ligands **1**–**6** and Gal-1 or Gal-3 (total volume: 50 μL; 4.5 μM final galectin concentration) in EPBS buffer were added and incubated for 2 h. Each step was followed by extensive rinsing of the wells with Tween 20 (0.05% *v*/*v*) in PBS. Bound galectins were detected with anti-His_6_-IgG1 mouse antibody conjugated to horseradish peroxidase (Roche Diagnostics, Mannheim, Germany) in PBS (1/500 for Gal-1 and 1/1000 for Gal-3, 1 h, r.t., 50 μL/well). The IgG-conjugated peroxidase was quantified using TMB One substrate (Kem-En-Tec, Taastrup, Denmark). The reaction was stopped by adding 3 M HCl (50 μL) for colorimetric detection at 450 nm.

### 3.5. Galectin Expression

#### 3.5.1. Expression of Gal-1 and Gal-3 CRD Unlabeled

The expression protocol is common for Gal-1 and Gal-3 CRD. The gene encoding the sequence of Gal-1 (135 residues) was inserted into the pET21a expression vector. The gene encoding the carbohydrate recognition domain (CRD) of Gal-3 containing 138 residues was inserted into the pET21a expression vector. BL21(D3) *E. coli* competent cells were transformed with the respective expression vector using the following heat shock method: 42 °C for 90 s and subsequently 5 min on ice. After 1 night of incubation on agar plates at 37 °C in the presence of the antibiotic ampicillin at a concentration of 100 μg/mL, a colony harboring the expression construct was selected and then inoculated with 200 mL Luria Broth (LB) medium containing 100 μg/mL ampicillin overnight at 37 °C with shaking. The precise amount of the grown culture to achieve a final OD_600_ of 0.1 in 2 L of fresh LB medium containing ampicillin was added. After that, the culture was grown at 37 °C until *OD*_600_ reached 0.6–1.2. Then, the culture was induced with 1 mM isopropyl 1-thio-β-d-galactopyranoside (IPTG). At 3 h after induction, the induced culture was harvested by centrifugation at 5500 rpm for 30 min. The pellet was purified as explained in the respective section.

#### 3.5.2. Expression of Gal-1 and Gal-3 CRD 15N Labeled

The expression protocol was common for Gal-1 and Gal-3 CRD. The gene encoding the sequence of Gal-1 (135 residues) was inserted into the pET21a expression vector. The gene encoding the carbohydrate recognition domain (CRD) of Gal-3 CRD containing 138 residues was inserted into the pET21a expression vector. The BL21 (D3) *E. coli* competent cells were transformed with the respective expression vector using heat shock method: 42 °C for 90 s and then 5 min on ice. After 1 night of incubation on agar plates at 37 °C in the presence of the antibiotic ampicillin at a concentration of 100 μg/mL, a colony harboring the expression construct was selected and then inoculated with 5 mL of Luria Broth (LB) medium containing 100 μg/mL ampicillin for 6 h at 37 °C with shaking. The culture was centrifuged at 4400 rpm for 5 min ant the pellet was resuspended in 1 mL of M9 minimum medium containing ampicillin and transferred in a flask with 200 mL of the same medium. The flask was then incubated overnight at 37 °C with shaking. The precise amount of the grown culture to achieve a final *OD*_600_ of 0.1 in 2 L of fresh M9-labeled (^15^N-NH_4_Cl as nitrogen source) medium containing ampicillin was added. After that, the culture was grown at 37 °C until *OD*_600_ reached 0.6–1.2. Then, the culture was induced with 1 mM IPTG. At 3 h after induction, the induced culture was harvested by centrifugation at 5500 rpm for 30 min. The pellet was purified as explained in the respective section.

### 3.6. Galectin Purification

The purification protocol was common for Gal-1 and Gal-3. The only difference was the addition of the reducing agent DTT in some buffers for the purification of Gal-1, justified by the presence of cysteine residues exposed to the solvent that could cause the formation of nonspecific dimers or aggregates through intermolecular disulfide bonds.

The pellet obtained from the centrifugation of the 2 L suspension of BL21 cells was suspended in lysis buffer composed as follows: 22 mM Tris-HCl, 5 mM EDTA, 1 mM PMSF, and 1 mM DTT (only in the case of Gal-1) at pH 7.5. The cell suspension was left for 30 min on ice with shaking and then lysed by sonication on ice with 60% amplitude, 12 repetitions of 20 s, and 59 s of an interval between each burst. The extract was clarified by ultracentrifugation at 35,000 rpm for 1 h at 4 °C. The soluble fraction was loaded onto 5 mL of α-lactose-agarose resin (Sigma-Aldrich, Prague, Czech Republic) which was already equilibrated with equilibration buffer (50 mM TRIS pH 7.2, 150 mM NaCl). The protein was eluted with 7 mL of elution buffer (150 mM lactose pH 7.4 in PBS 1×). Lectin purity was checked by 4–12% SDS-PAGE and by LC-MS. To eliminate lactose from the samples, a series of dialysis and washes with centrifuge filters (Sartorius Vivaspin 6 5000 MWCO, Sartorius, Göttingen, Germany) using fresh buffer (50 mM sodium phosphate, 150 mM NaCl, and 2 mM DTT in the case of Gal-1, pH 7.4) was performed. The absence of lactose was checked by 1D NMR. The sample of Gal-1 and Gal-3 CRD used for cryo-microscopy were further purified with size-exclusion chromatography using a Superdex 10/300 75 Increase column and the elution peak corresponding to the dimeric and monomeric form of the lectin, respectively, was collected and concentrated for the following analysis. 

### 3.7. NMR Spectroscopy

^1^H NMR spectra of ligands **2**–**6** were acquired using a Bruker AVANCE III 600 MHz spectrometer (operating at 600.2 MHz) in CD_3_OD (for ligand **2**) or in D_2_O (for ligands **3**–**6**). The conditions applied for recording the spectra have previously been reported [24].

#### 3.7.1. Saturation Transfer Difference (STD) NMR

The STD experiments were acquired using the Bruker AVANCE 2 600 MHz spectrometer equipped with a 5 mm QCI cryo-probe (Bruker Inc.; Billerica, MA, USA). The samples (500 μL total in 5 mm standard NMR tubes) were prepared in deuterated phosphate saline buffer (50 mM sodium phosphate, 150 mM NaCl, pH 7.4). In total, 2 mM d,l-dithiothreitol-*d*_10_ (DTT-*d*_10_) was added to the buffer of the sample containing Gal-1. The standard ratio ligand/lectin was set at 1:30, with Gal-1 employed at a concentration of 100 μM and Gal-3 at 50 μM. During the acquisition, the temperature was set at 298 K. The STD sequence stddiffesgp.3 was selected from the Bruker library and included spoil and T2 filter using excitation sculpting. The off-resonance frequency was set at 100 ppm and the on-resonance frequency was set at 0.00 ppm, −0.5 ppm, or −1 ppm. The experiments were acquired using a train of 50 ms Gaussian-shaped pulses, 2 s of relaxation delay, and 1024 scans. To remove the NMR signals of the lectin, a spinlock filter of 30 ms was applied. The STD spectra were obtained by subtracting the on-resonance spectrum from the off-resonance spectrum. The STD Amplification Factor (STD-AF) was calculated based on the comparison between the signals of the STD spectrum and those of the off-resonance spectrum. The STD% was calculated by normalization of the whole set of STD factors against the highest value for each ligand (100% of STD effect) [28,29,30]. 

#### 3.7.2. Chemical Shift Perturbation (CSP) Analysis

The ^1^H-^15^N HSQC experiments were acquired using the Bruker AVANCE 2 600 MHz spectrometer equipped with a 5 mm QCI cryo-probe (Bruker Inc.; Billerica, MA, USA). All the samples (500 μL total in 5 mm standard NMR tubes) were prepared with ^15^N labeled lectin at a concentration of 100 μM for Gal-1 and 50 μM for Gal-3. The buffer used was 90% phosphate-buffered saline (50 mM sodium phosphate, 150 mM NaCl, pH 7.4) and 10% deuterated water (D_2_O). Next, 2 mM of DTT-*d*_10_ was added to the buffer of the sample containing Gal-1. During the acquisition, the temperature was set at 298 K.

Ligands **1**–**3** were titrated to the protein sample, and a ^1^H-^15^N HSQC experiment was recorded at each point. In total, 0.1-0.25-0.5-1-2.5-5 equivalents of ligands **1**–**3** were titrated to each lectin. The ^1^H-^15^N HSQC experiments were performed using the standard sequence from the Bruker library, with 192 (T1) and 2048 (T2) complex data points for ^15^N and ^1^H dimensions and 64 scans. The analysis of the chemical shift perturbation of the amide cross-peaks during the titration with the ligands, as well as the following of the intensities during the titration, was performed using CcpNMR Analysis software. The CSP data were obtained by applying the formula Δ*δ* (ppm) = [(Δ*δ*H2 + (0.14 · Δ*δ*N)2)/2] [31].

The plot with the results represents the Δ*δ* for each NH residue of the backbone of the lectins’ residues. The intensity plots were obtained using the formula *I*_i_-*I*_f_/Δ*I*_max_ for each cross-peak, respectively. 

Ligands **4**–**6** were titrated to the protein sample and a ^1^H-^15^N HSQC experiment was recorded at each point. In total, 1.5 equivalents of ligands **4**–**6** were added to Gal-1, and 4–5 equivalents of **4**–**6** were added to Gal-3. The recovery of the signals was performed by adding 5-10-20-50-75 equivalents of LacNAc competitor (**1**) to Gal-1 samples and 1-5-10-15 equivalents of the same competitor to Gal-3 samples. The ^1^H-^15^N HSQC experiments were performed using the standard sequence from the Bruker library, with 256 (T1) and 1536 (T2) complex data points for ^15^N and ^1^H dimension and 32 scans. The analysis of the total intensity of the spectra was made using CcpNMR Analysis software. The total intensities were normalized to the maximum intensity of the spectra with the lectin alone (100%).

### 3.8. Cryo-EM Sample Preparation and Data Collection

Cryo-EM experiments were performed by pipetting 4 µL of each sample onto R2/2 cupper 300-mesh (Quantifoil) grids. In the control samples, Gal-1 and Gal-3 protein concentration was 0.5 mg/mL, while ligand **4** was vitrified at 0.17 mg/mL. The mixture of protein and ligand was prepared following the ratio 5:1 (galectin/ligand) to obtain a final concentration of 0.5 mg/mL for Gal-3 and 0.17 mg/mL for ligand **4**, and 0.25 mg/mL for Gal-1 and 0.13 mg/mL for ligand **4**. In all cases, the buffer used was PBS 1× with pH 7.4. Prior to vitrification, grids were plasma-cleaned using the BAL-TEC MED 020 coating system. The samples were vitrified using the Leica EM GP2 plunge freezer to preincubate the sample in the chamber at 95% of humidity and at 8 °C for 30 s (blotting conditions: 1.5 s and 43 mm of offset). 

Images were acquired in-house with a JEM-2200FS/CR (JEOL Ltd., Tokyo, Japan) electron microscope, operating at 200 kV at liquid nitrogen temperature, and equipped with a K2 Summit direct detection camera (Gatan, Inc., Pleasanton, CA, USA). Dose fractionated movies were recorded with the Gatan DigitalMicrograph^TM^ software and motion correction of frames was performed within the same software. The movies were collected at a defocus range from −1.8 µm to −3.0 µm with a final dose of ~40 e–/Å^2^ at a nominal magnification of 30,000×, producing a pixel size of 1.28 Å at the specimen.

### 3.9. DLS Measurements

Particle size was measured by quasi-elastic light scattering using a Malvern Nano-S Zeta-Sizer spectrometer (Malvern Instruments, Worcestershire, UK). Standard acryl-cuvettes were employed. The samples were suspended in PBS 1× buffer pH 7.4, and all the experiments were acquired at room temperature. Galectins alone were tested at a concentration of 0.5 mg/mL, and ligand **4** was tested at a concentration of 1.5 mg/mL. For the mixtures, the lectin/ligand ratio, 5:1, was maintained in both cases.

## 4. Conclusions

The interaction of a variety of glycopolymers with two human galectins (Gal-1 and Gal-3) has been scrutinized by different techniques, including ligand- and receptor-based NMR, DLS, and electron microscopy methods. STD-NMR (from the glycan perspective) and ^1^H-^15^N HSQC (from the galectin perspective) experiments, titrating the lectins with increasing amounts of the glycans and glycopolymers, were carried out. The analysis of the NMR results shows that the glycopolymers and their constituting units, which present LacNAc moieties as interaction points, are recognized by the galectins in a canonical manner through their carbohydrate-binding sites, as further demonstrated through NMR-based competition experiments. Moreover, the analysis of the ^1^H-^15^N HSQC NMR data demonstrates that the two galectins show distinct interaction features, as revealed by the large intensity losses observed in the HSQC spectra of Gal-1 upon the addition of the multivalent ligands in comparison to those observed for galectin-3. In fact, the HSQC NMR spectra suggest that the glycopolymers and Gal-1 generate large supramolecular entities. This hypothesis was unambiguously assessed through DLS and especially EM data, which showed the presence of cross-linked entities. For the presentations of the LacNAc molecules on bivalent and trivalent branches on the glycopolymers (**5**, **6**), no enhanced interactions were achieved in comparison to those obtained for the presentation of individually distributed LacNAc (**4**), revealing the importance of ligand density and orientation for the establishment of proper intermolecular contacts. The combination of different complementary techniques (ligand- and receptor-based NMR, DLS, EM) provides a detailed picture of the interaction event at different resolutions, from the atomic to the supramolecular levels.

## Figures and Tables

**Table 1:** Physicochemical characteristics of glycopolymer ligands **4**–**6** *^a^*.

Ligand	LacNAc Presentation	LacNAc Content [mol.%] *^b^*	*M*_n_ [g mol^−1^] *^c^*	*M*_w_ [g mol^−1^] *^c^*	*Đ ^c^*
**4**	Individual	19.3	25,200	33,600	1.3
**5**	bivalent branching	20.9	30,200	37,900	1.3
**6**	trivalent branching	22.0	32,400	38,400	1.2

*^a^* Data were adopted from our previous work [24]. *^b^* NMR (600.2 MHz) was used to determine the content of LacNAc using D_2_O as solvent. *^c^* SEC was employed to determine the number-average molecular weight (*M*_n_), the weight-average molecular weight (*M*_w_), and the dispersity (*Đ*) using RI and MALS detectors. Superose 6 column was used with 0.3 M sodium acetate buffer (pH 6.5) as a mobile phase.

**Table 2:** The I*C*_50_ (μM) and relative potency per active unit (*rp*/*n*) for **1**–**6** obtained with ASF-competitive ELISA assay ^a^.

	Ligand 1	Ligand 2	Ligand 3	Ligand 4	Ligand 5	Ligand 6
Gal-1	78 ± 23	19 ± 5	9 ± 3	0.086 ± 0.05	0.41 ± 0.08	0.082 ± 0.02
*rp*/*n*	1	2	2.8	906	95	317
Gal-3	44 ± 8	12 ± 2	4.4 ± 1.6	11 ± 1	4.6 ± 1.3	1.7 ± 0.4
*rp*/*n*	1	1.8	3.3	4	4.8	8.6

^a^ The data were adopted from our previous work [24].

## Data Availability

The data presented in this study are available on request from the corresponding authors.

## Supplementary Materials

The Supplementary Materials are available online at https://www.mdpi.com/article/10.3390/ijms22116000/s1.

## References

[1] Laaf D., Bojarová P., Elling L., Křen V. (2019). Galectin–Carbohydrate Interactions in Biomedicine and Biotechnology. Trends Biotechnol..

[2] Ebrahim A.H., Alalawi Z., Mirandola L., Rakhshanda R., Dahlbeck S., Nguyen D., Jenkins M., Grizzi F., Cobos E., Figueroa J.A. (2014). Galectins in cancer: Carcinogenesis, diagnosis and therapy. Ann. Transl. Med..

[3] Cousin J., Cloninger M. (2016). The Role of Galectin-1 in Cancer Progression, and Synthetic Multivalent Systems for the Study of Galectin-1. Int. J. Mol. Sci..

[4] Nangia-Makker P., Balan V., Raz A. (2008). Regulation of tumor progression by extracellular galectin-3. Cancer Microenviron..

[5] Dings R., Miller M., Griffin R., Mayo K. (2018). Galectins as Molecular Targets for Therapeutic Intervention. Int. J. Mol. Sci..

[6] Stillman B.N., Hsu D.K., Pang M., Brewer C.F., Johnson P., Liu F.-T., Baum L.G. (2006). Galectin-3 and Galectin-1 Bind Distinct Cell Surface Glycoprotein Receptors to Induce T Cell Death. J. Immunol..

[7] Girard A., Magnani J.L. (2018). Clinical Trials and Applications of Galectin Antagonists. Trends Glycosci. Glycotechnol..

[8] Bertuzzi S., Quintana J.I., Ardá A., Gimeno A., Jiménez-Barbero J. (2020). Targeting Galectins with Glycomimetics. Front. Chem..

[9] Rabinovich G.A., Cumashi A., Bianco G.A., Ciavardelli D., Iurisci I., D’Egidio M., Piccolo E., Tinari N., Nifantiev N., Iacobelli S. (2006). Synthetic lactulose amines: Novel class of anticancer agents that induce tumor-cell apoptosis and inhibit galectin-mediated homotypic cell aggregation and endothelial cell morphogenesis. Glycobiology.

[10] Laaf D., Steffens H., Pelantová H., Bojarová P., Křen V., Elling L. (2017). Chemo-Enzymatic Synthesis of Branched *N*-Acetyllactosamine Glycan Oligomers for Galectin-3 Inhibition. Adv. Synth. Catal..

[11] Laaf D., Bojarová P., Pelantová H., Křen V., Elling L. (2017). Tailored Multivalent Neo-Glycoproteins: Synthesis, Evaluation, and Application of a Library of Galectin-3-Binding Glycan Ligands. Bioconjug. Chem..

[12] Zhang H., Laaf D., Elling L., Pieters R.J. (2018). Thiodigalactoside-Bovine Serum Albumin Conjugates as High-Potency Inhibitors of Galectin-3: An Outstanding Example of Multivalent Presentation of Small Molecule Inhibitors. Bioconjug. Chem..

[13] Soomro Z.H., Cecioni S., Blanchard H., Praly J.P., Imberty A., Vidal S., Matthews S.E. (2011). CuAAC synthesis of resorcin[4]arene-based glycoclusters as multivalent ligands of lectins. Org. Biomol. Chem..

[14] Sakamoto J.I., Koyama T., Miyamoto D., Yingsakmongkon S., Hidari K.I.P.J., Jampangern W., Suzuki T., Suzuki Y., Esumi Y., Hatano K. (2007). Thiosialoside clusters using carbosilane dendrimer core scaffolds as a new class of influenza neuraminidase inhibitors. Bioorg. Med. Chem. Lett..

[15] Nagahori N., Nishimura S.-I. (2006). Direct and Efficient Monitoring of Glycosyltransferase Reactions on Gold Colloidal Nanoparticles by Using Mass Spectrometry. Chem. Eur. J..

[16] Heine V., Hovorková M., Vlachová M., Filipová M., Bumba L., Janoušková O., Hubálek M., Cvačka J., Petrásková L., Pelantová H. (2021). Immunoprotective neo-glycoproteins: Chemoenzymatic synthesis of multivalent glycomimetics for inhibition of cancer-related galectin-3. Eur. J. Med. Chem..

[17] Gimeno A., Delgado S., Valverde P., Bertuzzi S., Berbís M.A., Echavarren J., Lacetera A., Martín-Santamaría S., Surolia A., Cañada F.J. (2019). Minimizing the Entropy Penalty for Ligand Binding: Lessons from the Molecular Recognition of the Histo Blood-Group Antigens by Human Galectin-3. Angew. Chem. Int. Ed..

[18] Bertuzzi S., Gimeno A., Núñez-Franco R., Bernardo-Seisdedos G., Delgado S., Jiménez-Osés G., Millet O., Jiménez-Barbero J., Ardá A. (2020). Unravelling the Time Scale of Conformational Plasticity and Allostery in Glycan Recognition by Human Galectin-1. Chem. Eur. J..

[19] Ardá A., Jiménez-Barbero J. (2018). The recognition of glycans by protein receptors. Insights from NMR spectroscopy. Chem. Commun..

[20] Ulbrich K., Holá K., Šubr V., Bakandritsos A., Tuček J., Zbořil R. (2016). Targeted Drug Delivery with Polymers and Magnetic Nanoparticles: Covalent and Noncovalent Approaches, Release Control, and Clinical Studies. Chem. Rev..

[21] Chytil P., Koziolová E., Etrych T., Ulbrich K. (2018). HPMA Copolymer-Drug Conjugates with Controlled Tumor-Specific Drug Release. Macromol. Biosci..

[22] Filipová M., Bojarová P., Rodrigues Tavares M., Bumba L., Elling L., Chytil P., Gunár K., Křen V., Etrych T., Janoušková O. (2020). Glycopolymers for Efficient Inhibition of Galectin-3: In Vitro Proof of Efficacy Using Suppression of T Lymphocyte Apoptosis and Tumor Cell Migration. Biomacromolecules.

[23] Bojarová P., Tavares M.R., Laaf D., Bumba L., Petrásková L., Konefał R., Bláhová M., Pelantová H., Elling L., Etrych T. (2018). Biocompatible glyconanomaterials based on HPMA-copolymer for specific targeting of galectin-3. J. Nanobiotechnol..

[24] Tavares M.R., Bláhová M., Sedláková L., Elling L., Pelantová H., Konefał R., Etrych T., Křen V., Bojarová P., Chytil P. (2020). High-Affinity *N*-(2-Hydroxypropyl) methacrylamide Copolymers with Tailored *N*-Acetyllactosamine Presentation Discriminate between Galectins. Biomacromolecules.

[25] Bojarová P., Kulik N., Hovorková M., Slámová K., Pelantová H., Křen V. (2019). The β-*N*-Acetylhexosaminidase in the Synthesis of Bioactive Glycans: Protein and Reaction Engineering. Molecules.

[26] Mészáros Z., Nekvasilová P., Bojarová P., Křen V., Slámová K. (2021). Advanced glycosidases as ingenious biosynthetic instruments. Biotechnol. Adv..

[27] Gimeno A., Valverde P., Ardá A., Jiménez-Barbero J. (2020). Glycan structures and their interactions with proteins. A NMR view. Curr. Opin. Struct. Biol..

[28] Mayer M., Meyer B. (1999). Characterization of ligand binding by saturation transfer difference NMR spectroscopy. Angew. Chem. Int. Ed..

[29] Meyer B., Peters T. (2003). NMR Spectroscopy Techniques for Screening and Identifying Ligand Binding to Protein Receptors. Angew. Chem. Int. Ed..

[30] Viegas A., Manso J., Nobrega F.L., Cabrita E.J. (2011). Saturation-transfer difference (STD) NMR: A simple and fast method for ligand screening and characterization of protein binding. J. Chem. Educ..

[31] Williamson M.P. (2013). Using chemical shift perturbation to characterise ligand binding. Prog. Nucl. Magn. Reson. Spectrosc..

[32] Šubr V., Ulbrich K. (2006). Synthesis and properties of new *N*-(2-hydroxypropyl) methacrylamide copolymers containing thiazolidine-2-thione reactive groups. React. Funct. Polym..

[33] Chytil P., Etrych T., Kříž J., Šubr V., Ulbrich K. (2010). *N*-(2-Hydroxypropyl) methacrylamide-based polymer conjugates with pH-controlled activation of doxorubicin for cell-specific or passive tumour targeting. Synthesis by RAFT polymerisation and physicochemical characterisation. Eur. J. Pharm. Sci..

[34] Bumba L., Laaf D., Spiwok V., Elling L., Křen V., Bojarová P. (2018). Poly-*N*-Acetyllactosamine Neo-Glycoproteins as Nanomolar Ligands of Human Galectin-3: Binding Kinetics and Modeling. Int. J. Mol. Sci..

