# Supplementary material for: Cross-Linking Effects Dictate the Preference of Galectins to Bind LacNAc-Decorated HPMA Copolymers
Source: Int J Mol Sci. 2021 Jun 1;22(11):6000. doi: 10.3390/ijms22116000 (PMC8199549; doi:10.3390/ijms22116000)
Supplement: Supplementary file 1 [file ijms-22-06000-s001.zip › ijms-1241031-supplementary.pdf]

SUPPLEMENTARY MATERIALS

NMR EXPERIMENTS

Saturation Transfer Difference (STD) NMR

All the STD experiments were acquired using AVANCE 2 600 MHz spectrometer equipped with a 5 mm QCI cryo-probe (Bruker Inc.; Billerica, MA, USA). The samples (500 µL total in 5mm standard NMR tubes) were prepared in deuterated phosphate saline buffer (50 mM sodium phosphate, 150 mM NaCl, pH 7.4 – 2 mM of dithiothreitol-*d*10 (DTT-*d*10) was added to the samples containing Gal-1). The STD spectra acquired with different irradiations and 2 s of saturation time for ligands **2** and **3** and Gal-1 and Gal-3-CRD are reported here, together with the relative STD Amplification Factor (STD-AF) intensities and STD percentage (STD%) calculated. The STD% values obtained from the spectra with the irradiation at δ -0.5 ppm were used for the representation of the epitope mapping (Figure S1 and main test).

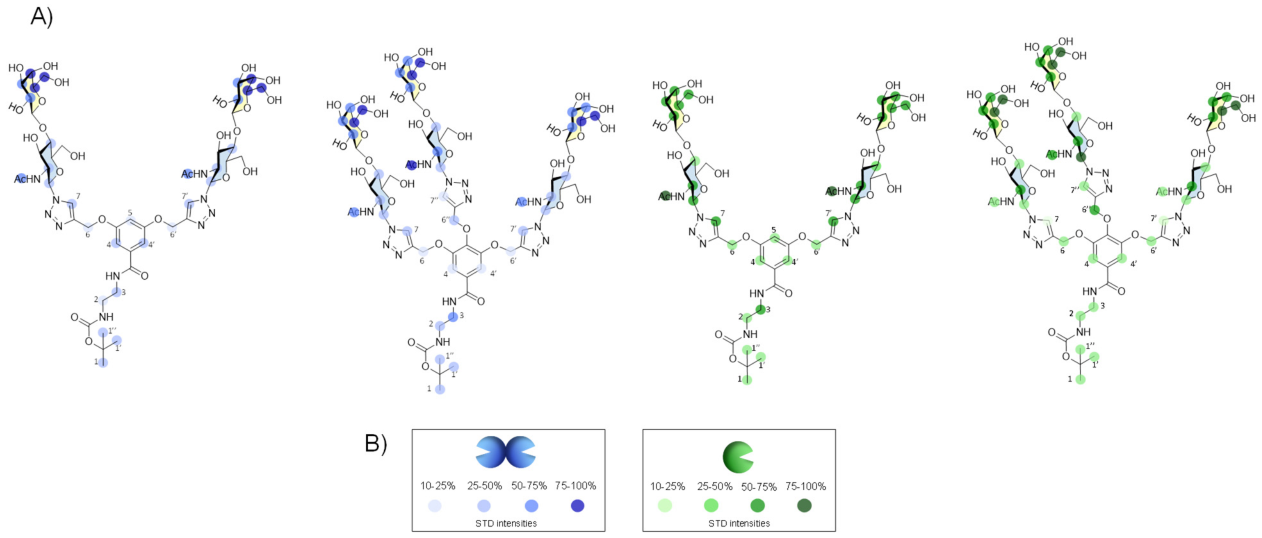

Figure S1. Binding epitopes of ligand 2 and 3 for Gal-1 and Gal-3 CRD. (**A**) STD binding epitopes for ligands **2** and **3** and Gal-1 (blue) and Gal-3 CRD (green) obtained from spectra acquired with irradiation at δ-0.5 ppm. (**B**) Color legend of the percentage of STD.

A)

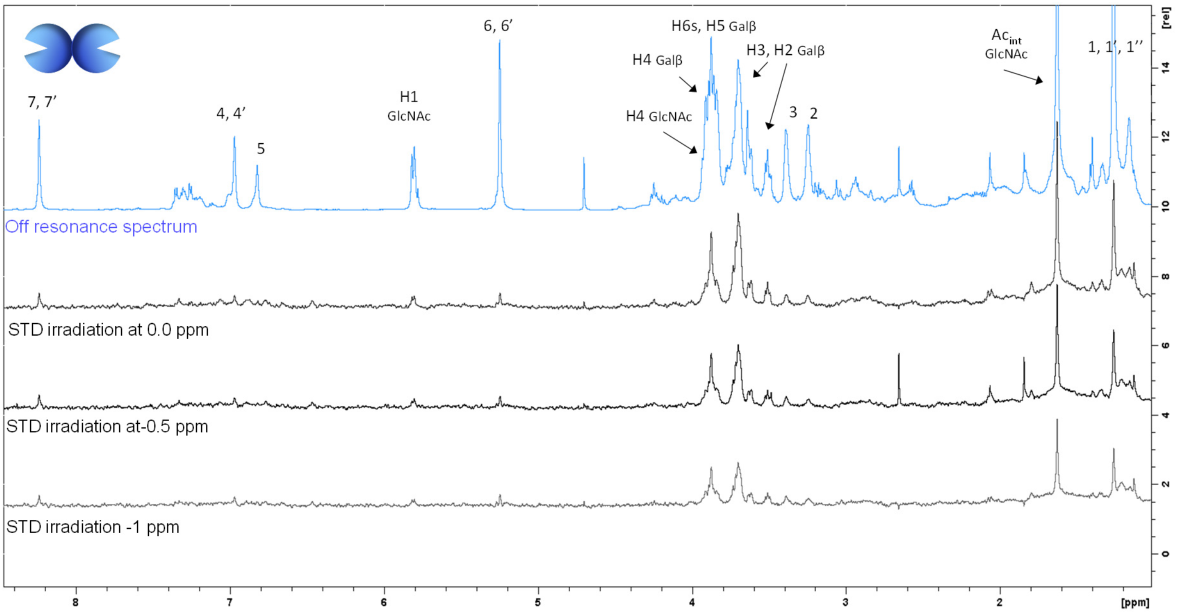

B)

0.0 ppm

Residue	Atom	STD-AF	STD %
GlcNAc	H1	0.014	30%
	H2	0.0232	49%
	H3	-	-
	H4	0.016	34%
	H5	-	-
	H6, H6'	-	-
	Ac	0.034	78%
Galβ	H1	-	-
	H2	0.03	63%
	H3	0.03	63%
	H4	0.038	80%
	H5	0.047	100%
	H6s	0.047	100%
scaffold	1, 1', 1''	0.02	42%
	2	0.01	21%
	3	0.012	25%
	4, 4'	0.012	25%
	5	0.011	23%
	6, 6'	0.006	12%
	7, 7'	0.012	25%

-0.5 ppm

Residue	Atom	STD-AF	STD %
GlcNAc	H1	0.0108	35%
	H2	0.0156	50%
	H3	-	-
	H4	0.0119	38%
	H5	-	-
	H6, H6'	-	-
	Ac	0.023	74%
Galβ	H1	-	-
	H2	0.022	71%
	H3	0.022	71%
	H4	0.024	77%
	H5	0.031	100%
	H6s	0.031	100%
scaffold	1, 1', 1''	0.012	38%
	2	0.008	25%
	3	0.01	32%
	4, 4'	0.01	32%
	5	0.008	25%
	6, 6'	0.005	16%
	7, 7'	0.01	32%

-1.0 ppm

Residue	Atom	STD-AF	STD %
GlcNAc	H1	0.0078	35%
	H2	0.011	50%
	H3	-	-
	H4	0.009	45%
	H5	-	-
	H6, H6'	-	-
	Ac	0.016	72%
Galβ	H1	-	-
	H2	0.016	72%
	H3	0.016	72%
	H4	0.017	77%
	H5	0.022	100%
	H6s	0.022	100%
scaffold	1, 1', 1''	0.008	36%
	2	0.007	31%
	3	0.009	40%
	4, 4'	0.009	45%
	5	0.007	31%
	6, 6'	0.005	22%
	7, 7'	0.009	45%

Figure S2. STD Gal-1 and ligand **2**. (**A**) Sample: Gal-1 100 μM, **2** 3 mM (ratio lectin:ligand = 1:30). Off-resonance (irradiation at 100 ppm) and STD spectra (irradiation at δ 0.0, δ -0.5 and δ -1 ppm, 50×). (**B**) Relative STD-AF intensities and STD percentage for each proton signal calculated on the basis of the relative experiment.

A)

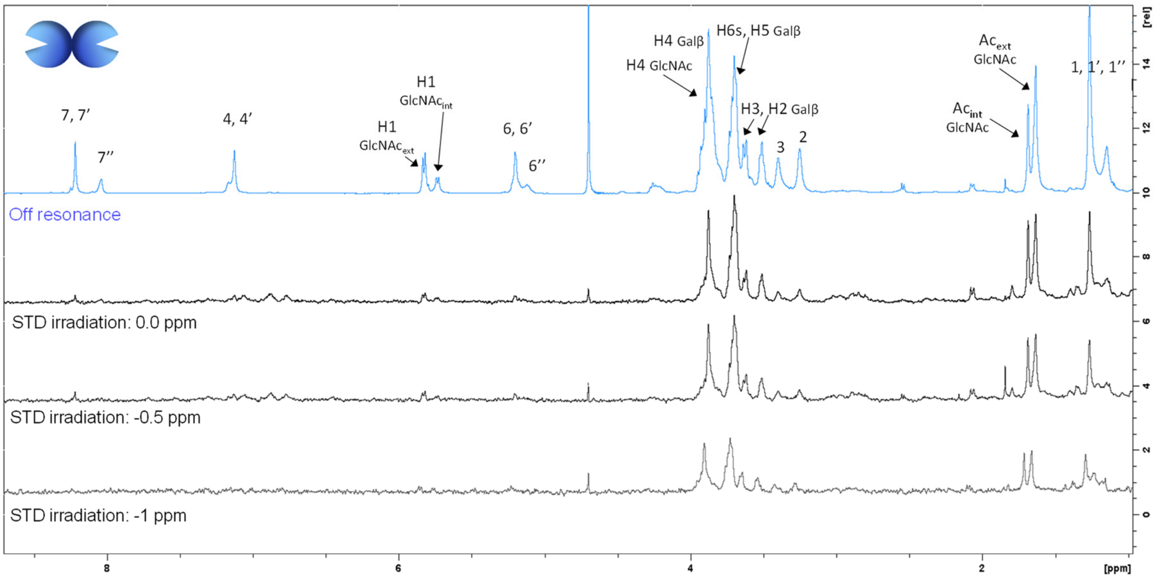

B)

0.0 ppm

Residue	Atom	STD-AF	STD %
GlcNAc	$H1_{ext}$	0.012	25%
	$H1_{int}$	0.017	35%
	$H2_{ext}$	0.02	42%
	$H2_{int}$	0.025	52%
	H3	-	-
	H4	0.017	35%
	H5	-	-
	H6, H6'	-	-
	Ac_{ext}	0.036	75%
	Ac_{int}	0.048	100%
Galβ	H1	-	-
	H2	0.03	62%
	H3	0.03	62%
	H4	0.034	70%
	H5	0.042	87%
	H6s	0.042	87%
scaffold	1, 1', 1''	0.023	47%
	2	0.015	31%
	3	0.016	33%
	4, 4'	0.0085	17%
	6, 6'	0.009	18%
	6''	0.01	20%
	7, 7'	0.0073	15%
	7''	0.012	25%

-0.5 ppm

Residue	Atom	STD-AF	STD %
GlcNAc	$H1_{ext}$	0.01	31%
	$H1_{int}$	0.014	45%
	$H2_{ext}$	0.015	48%
	$H2_{int}$	0.016	52%
	H3	-	-
	H4	0.012	38%
	H5	-	-
	H6, H6'	-	-
	Ac_{ext}	0.023	74%
	Ac_{int}	0.031	100%
Galβ	H1	-	-
	H2	0.019	61%
	H3	0.019	61%
	H4	0.02	64%
	H5	0.027	87%
	H6s	0.027	87%
scaffold	1, 1', 1''	0.013	42%
	2	0.012	38%
	3	0.019	61%
	4, 4'	0.007	22%
	6, 6'	0.0075	24%
	6''	0.009	29%
	7, 7'	0.007	22%
	7''	0.009	29%

-1 ppm

Residue	Atom	STD-AF	STD %
GlcNAc	$H1_{ext}$	0.005	31%
	$H1_{int}$	0.008	50%
	$H2_{ext}$	0.008	50%
	$H2_{int}$	0.009	56%
	H3	-	-
	H4	0.008	50%
	H5	-	-
	H6, H6'	-	-
	Ac_{ext}	0.011	68%
	Ac_{int}	0.016	100%
Galβ	H1	-	-
	H2	0.01	62%
	H3	0.01	62%
	H4	0.011	68%
	H5	0.015	93%
	H6s	0.015	93%
scaffold	1, 1', 1''	0.0066	41%
	2	0.007	43%
	3	0.008	50%
	4, 4'	0.0045	28%
	6, 6'	0.0045	28%
	6''	0.006	37%
	7, 7'	0.0025	15%
	7''	0.004	25%

Figure S3. STD Gal-1 and ligand **3**. (**A**) Sample: Gal-1 100 μM, **3** 3 mM (ratio lectin:ligand = 1:30). Off-resonance (irradiation at 100 ppm) and STD spectra (irradiation at δ 0.0, δ -0.5 and δ -1 ppm, 50×). (**B**) Relative STD-AF intensities and STD percentage for each proton signal calculated on the basis of the relative experiment.

A)

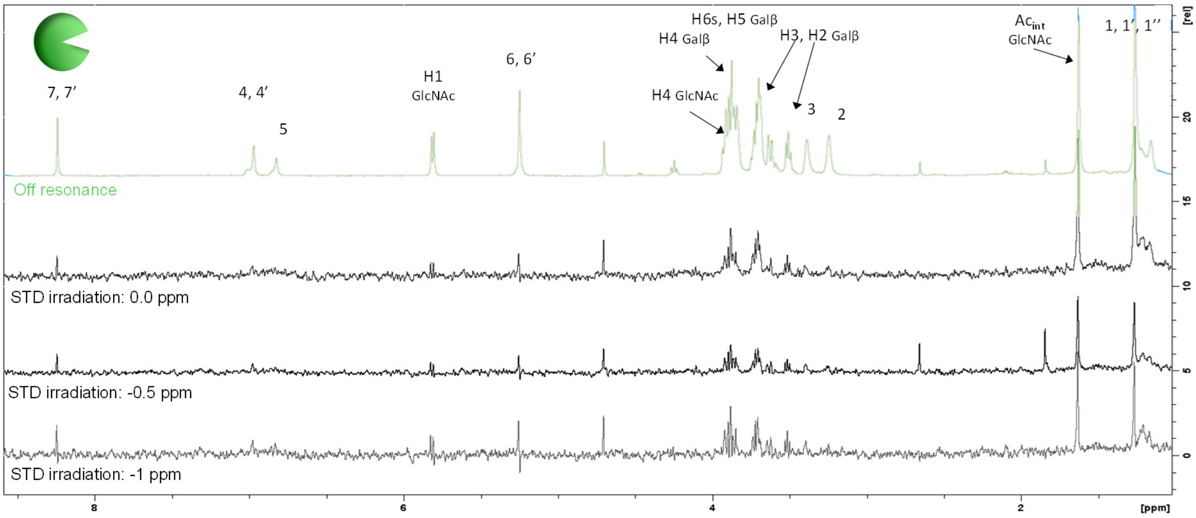

B)

0.0 ppm

Residue	Atom	STD-AF	STD %
GlcNAc	H1	0.008	42%
	H2	0.009	47%
	H3	-	-
	H4	0.007	36%
	H5	-	-
	H6, H6'	-	-
	Ac	0.019	100%
Galβ	H1	-	-
	H2	0.01	52%
	H3	0.009	47%
	H4	0.0095	50%
	H5	0.011	57%
	H6s	0.011	57%
scaffold	1, 1', 1''	0.015	78%
	2	0.006	31%
	3	0.007	36%
	4, 4'	0.008	42%
	5	0.01	52%
	6, 6'	0.006	31%
	7, 7'	0.008	42%

-0.5 ppm

Residue	Atom	STD-AF	STD %
GlcNAc	H1	0.0087	60%
	H2	0.009	62%
	H3	-	-
	H4	0.0069	48%
	H5	-	-
	H6, H6'	-	-
	Ac	0.0143	100%
Galβ	H1	-	-
	H2	0.0098	68%
	H3	0.0082	57%
	H4	0..0077	53%
	H5	0.0085	59%
	H6s	0.0085	59%
scaffold	1, 1', 1''	0.0105	73%
	2	0.0055	38%
	3	0.0078	54%
	4, 4'	0.01	69%
	5	0.011	76%
	6, 6'	0.0066	46%
	7, 7'	0.01	76%

-1.0 ppm

Residue	Atom	STD-AF	STD %
GlcNAc	H1	0.009	75%
	H2	0.01	83%
	H3	-	-
	H4	0.0075	62%
	H5	-	-
	H6, H6'	-	-
	Ac	0.012	100%
Galβ	H1	-	-
	H2	0.01	83%
	H3	0.008	66%
	H4	0..008	66%
	H5	0.008	66%
	H6s	0.008	66%
scaffold	1, 1', 1''	0.0085	70%
	2	0.0050	41%
	3	0.0075	62%
	4, 4'	0.01	83%
	5	0.012	100%
	6, 6'	0.0077	64%
	7, 7'	0.01	83%

Figure S4. STD Gal-3-CRD and ligand **2**. (**A**) Sample: Gal-3-CRD 50 μM, **2** 1.5 mM (ratio lectin:ligand = 1:30). Off-resonance (irradiation at 100 ppm) and STD spectra (irradiation at δ 0.0, δ -0.5 and δ -1 ppm, 50×). (**B**) Relative STD-AF intensities and STD percentage for each proton signal calculated on the basis of the relative experiment.

A)

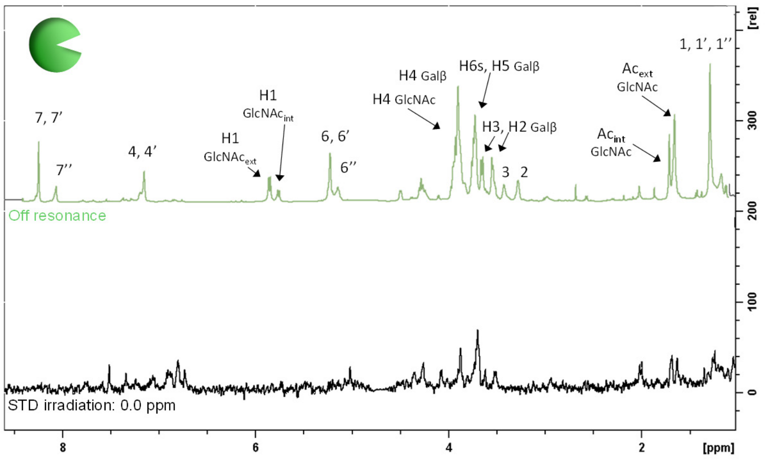

B)

-0.5 ppm

Residue	Atom	STD-AF	STD %
GlcNAc	H1$_{ext}$	0.004	44%
	H1$_{int}$	0.009	100%
	H2$_{ext}$	0.0066	73%
	H2$_{int}$	0.0066	73%
	H3	-	-
	H4	0.003	33%
	H5	-	-
	H6, H6'	-	-
	Ac$_{ext}$	0.004	44%
	Ac$_{int}$	0.0066	73%
Galβ	H1	-	-
	H2	0.006	66%
	H3	0.0066	73%
	H4	0.0045	50%
	H5	0.0085	94%
	H6s	0.0085	94%
scaffold	1, 1', 1''	0.0025	27%
	2	0.003	33%
	3	0.003	33%
	4, 4'	0.0028	31%
	6, 6'	0.0028	31%
	6''	0.005	55%
	7, 7'	0.0016	17%
	7''	0.004	44%

Figure S5. STD Gal-3-CRD and ligand **3**. (**A**) Sample: Gal-3-CRD 50 μM, **3** 1.5 mM (ratio lectin:ligand = 1:30). Off-resonance (irradiation at 100 ppm) and STD spectrum (irradiation at δ -0.5, 50×). (**B**) Relative STD-AF intensities and STD percentage for each proton signal calculated on the basis of the experiment.

DYNAMIC LIGHT SCATTERING MEASUREMENTS

Gal-1 0.5 mg/ml 4.54 nm + 0.6
Gal-3 0.5 mg/ml 1.56 nm + 0.19
Ligand **4** 1.5 mg/ml 7.47 nm + 1.8

Gal-1 0.5 mg/ml + Ligand **4** 1.5 mg/mL 875.0 nm + 128.1
Gal-3 0.5 mg/ml + Ligand **4** 1.5 mg/mL 8.43 nm + 1.23

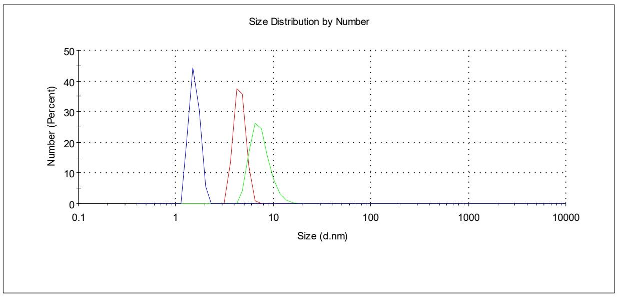

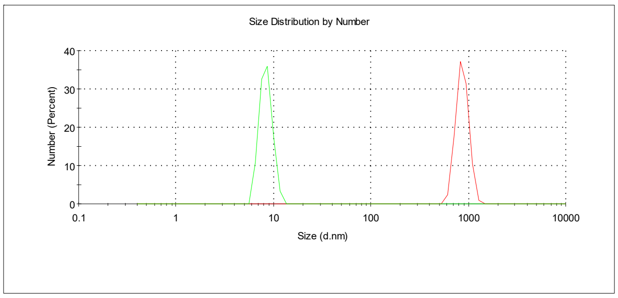

Figure S6. Hydrodynamic radius distribution by number measurements by dynamic light scattering. To the left, hydrodynamic radius distribution graphs for proteins Gal-1 and Gal-3-CRD at 0.5 mg/ml and ligand **4** at 1.5 mg/ml, indicating homogeneity by number distribution; peaks from multiple measurements indicate highly pure and homogenous preparations. To the right, hydrodynamic radius distribution graphs for ligand **4** complexed with Gal-3-CRD and Gal-1. Homogenous peaks are observed at different sizes, around 9 nm for ligand **4**–Gal-3 CRD complex and around 900 nm for ligand **4** – Gal-1 CRD complex.
